# Developing Potential Candidates of Preclinical Preeclampsia

**DOI:** 10.3390/ijms161126023

**Published:** 2015-11-13

**Authors:** Sandra Founds, Xuemei Zeng, David Lykins, James M. Roberts

**Affiliations:** 1 School of Nursing and Magee-Womens Research Institute, University of Pittsburgh, 3500 Victoria St. 448 VB, Pittsburgh, PA 15261, USA; 2 Biomedical Mass Spectrometry Center Schools of the Health Sciences, University of Pittsburgh, Biomedical Science Tower 3, 3501 Fifth Avenue, Pittsburgh, PA 15261, USA; xuz2@pitt.edu; 3 Magee-Womens Research Institute, University of Pittsburgh, 204 Craft Avenue Pittsburgh, PA 15213, USA; lykinsd@upmc.edu; 4 School of Medicine, Graduate School of Public Health and Magee-Womens Research Institute, University of Pittsburgh, 204 Craft Avenue Pittsburgh, PA 15213, USA; jroberts@mwri.magee.edu

**Keywords:** preeclampsia, candidate gene, candidate protein, biomarker development, prevention

## Abstract

The potential for developing molecules of interest in preclinical preeclampsia from candidate genes that were discovered on gene expression microarray analysis has been challenged by limited access to additional first trimester trophoblast and decidual tissues. The question of whether these candidates encode secreted proteins that may be detected in maternal circulation early in pregnancy has been investigated using various proteomic methods. Pilot studies utilizing mass spectrometry based proteomic assays, along with enzyme linked immunosorbent assays (ELISAs), and Western immunoblotting in first trimester samples are reported. The novel targeted mass spectrometry methods led to robust multiple reaction monitoring assays. Despite detection of several candidates in early gestation, challenges persist. Future antibody-based studies may lead to a novel multiplex protein panel for screening or detection to prevent or mitigate preeclampsia.

## 1. Introduction

Preeclampsia continues to be one of three leading causes of maternal mortality worldwide [1]. Mothers and babies who survive preeclampsia suffer serious morbidity and are at increased risk of later life cardiovascular diseases [2,3]. Preeclampsia is diagnosed at the new onset of hypertension and proteinuria after mid-gestation or new onset of hypertension without proteinuria but with presence of thrombocytopenia, renal, liver, pulmonary, or cerebral abnormalities [4].

Prediction, prevention, early detection, and personalized treatment of preeclampsia have been stymied by an incomplete understanding of its etiopathogenesis. This genetically complex disorder involves mother, fetus, and their interactions, while at the same time, preeclampsia is multifactorial and seems furthermore to consist of various subsets such as early and late onset [5,6]. One characteristic feature, however, around which theories and sub-classifications of preeclampsia revolve is the placenta. The placenta is necessary and sufficient to establish preeclampsia; consequently, delivery continues to be the only known cure [7,8].

Based on the centrality of the placenta to preeclampsia origins, a global gene expression microarray analysis was undertaken to identify genes and pathways early in preclinical disease and unbiased by candidate-specific hypotheses [7]. Currently, it is thought that the pathogenesis of preeclampsia includes reduced trophoblast invasion in the first and early second trimesters leading to shallow placentation and the failed remodeling of maternal spiral arteries supplying the placental site [9,10]. To begin to identify pertinent molecules in these maternal-fetoplacental processes, placental and decidual cells from women obtained as residual tissue from diagnostic chorionic villus sampling (CVS) procedures were analyzed. Assumptions were that effects of disease on gene expression would be lower at earlier gestational ages, and that maternal as well as trophoblast contributions could be elucidated. Samples of women who later developed preeclampsia and others with uncomplicated pregnancies were compared, resulting in a panel of 36 high priority preeclampsia candidate genes at 10–12 weeks of gestation [7].

Obtaining additional surplus CVS specimens poses a critical barrier to further examining this group of novel first trimester genomic candidates as potential biomarkers of preclinical preeclampsia. The use of fluorescent *in situ* hybridization (FISH) for fetal karyotyping has required less tissue by CVS and increased use of noninvasive prenatal genetic screening has reduced the numbers of CVS procedures. Nonetheless, alternative strategies have yielded support for the panel of 36 candidate genes. Remaining mRNA from the microarray samples were submitted to quantitative polymerase chain reaction (qPCR) studies, demonstrating *LAIR2* and *FSTL3* were each lower with large effect size in preeclampsia compared with uncomplicated pregnancy, which was consistent with the microarray results [11]. Additionally, there was no anti-correlation among the other investigated candidates by qPCR compared with the microarray data [11]. A review of literature associated 36 percent of the preeclampsia candidates with large population linkage studies and genome scans [12]. Selected candidates that were genotyped with samples independent of the CVS microarray study found differences between cases and controls in *ELL2*, *F11R*, *FN1*, and *SEMA3C* [13]. The genotypes may contribute to differential candidate gene expression.

In order to further explore the 36 preeclampsia candidates in more accessible, noninvasively obtained tissue, proteomics pilot studies have been conducted as another approach toward clinical translation. The purpose of this report is to present preliminary data based on the question of whether proteins encoded by these preeclampsia candidates can be quantified in maternal circulation during the first trimester of pregnancy. To investigate the potential of translating our findings from tissue mRNA expression discovery results to a blood based assay for early identification of preeclampsia, we applied three different approaches to detect and/or quantify the level of the expressed proteins of some of the 36 candidate genes [7] in maternal blood samples. Although these pilot studies met with limited success, the methods developed for our candidate-targeted assays may be of interest in comparison with other mass spectrometry studies directed at the discovery of proteomic candidates.

## 2. Results

### 2.1. LC-MS/MS MRM Assays

Our long range goals were to develop a multiplex panel for risk screening or preeclampsia detection prior to onset of signs and symptoms. Additionally, economizing on sample volumes of first trimester maternal blood with known pregnancy outcomes was a priority due to the labor intensive recruitment, collection, processing, and characterization of these prospective specimens in a disorder with a prevalence of 3% [4]. We piloted liquid chromatography-tandem mass spectrometry (LC-MS/MS) based multiple reaction monitoring (MRM) assay development to test for protein expression and to quantitate concentrations in maternal serum.

#### 2.1.1. Literature and Proteome Database Search

Although intervening posttranslational modifications occur [14], the possibility of these candidate genes producing proteins detectable in maternal circulation was supported by research literature and proteome databases. Table 1 demonstrates that at the time of the initial searches, a majority of the preeclampsia candidates were found to be expressed as proteins in normal fetoplacental tissues and/or maternal decidua, as well as in blood of pregnant and non-pregnant individuals. Limited numbers of enzyme-linked immunosorbent assays (ELISAs) were available at that time for most of the candidates and those available required relatively large volumes, e.g., 100–200 μL of sample per subject.

**Table 1:** Proteins encoded by preeclampsia candidate genes [7].

Gene Symbol_Dysregulation in Preeclampsia [7] *	Protein Name_UniProt Accession Number; Protein Expression in Normal Tissue	Blood_Concentration	ABs_Kits ^†^
CCK ↑	Cholecystokinin_P06307; Most normal tissues weak to moderate, gastrointestinal tract (GI) and a subset of neuronal cells strongly stained. Trophoblast (TB) moderate/Decidua negative (neg) [15]	1.3–4.2 pmol/L, 2nd trimester [16] 8.7 ± 1.2, 10.1 ± 1.6 and 10.4 ± 1.2 pM in 1st, 2nd, 3rd trimesters [17]	59_6
C4orf10 13.8 ↑	---**	-	-
S100A8 ↑	Protein S100-A8_P05109; Selective nuclear and cytoplasmic expression in squamous epithelia, subsets of cells outside reaction centra of lymphoid tissues and subsets of bone marrow poietic cells. TB neg/decidua neg [15]	2.7 × 10^5^ pg/mL Plasma [18]	33_6
CTAG2 ↑	Isoform LAGE-1B of Cancer/testis antigen 2 (LAGE-1L, Isoform LAGE-1A of Cancer/testis antigen 2 [LAGE-1S])_O75638-1, O75638-2; Peripheral blood mononuclear leukocytes [19]. Pending in The Human Protein Atlas (THPA) [15]	PBMCs [19]	8_0
CDNA: FLJ22732 8.4 ↑	---**	-	-
MUC15 -8.0 ↓	Isoform 1 of Mucin-15 (Cell membrane; Single-pass type I membrane protein), Isoform 2 of Mucin-15 (Secreted)_Q8N387-1, Q8N387-2; Normal tissues in general neg. Hepatocytes strong positivity. Strong staining in subset of cells in seminiferous ducts of testis. TB moderate/Decidua neg [15]	-	5_0
LOC440157 -8.1 ↓	Full-length cDNA 5-PRIME end of clone CS0DK007YB08 of HeLa cells of Homo sapiens (UniProt)_Q6NYL1	-	-
FN1 -8.2 ↓	Fibronectin (Isoforms 1 to 15)_P02751−1 to −15; Extracellular matrix, stromal cells, serum, bone marrow poetic cells and placenta show moderate to strong positivity. TB & Decidua moderate [15]	1.40 × 10^6^ pg/mL Plasma [18,20,21]; 11–43 μg/mL term gestational hypertension, PE, controls [21]	78_5
OXGR1 -8.2 ↓	2-Oxoglutarate receptor 1_Q96P68; Myometrium [19]. Pending THPA [15]	-	2_1
ELL2 -8.2 ↓	RNA polymerase II elongation factor ELL2_O00472; Most normal tissues moderate to strong positivity. Distinct membranous positivity in prostate, pancreatic islets, renal tubules. Intestine, bile ducts, cells in CNS, heart and skeletal muscle stained weak or neg. TB strong/Dedicua moderate [15]	-	8_0
SCARA5 -8.3 ↓	Scavenger receptor class A member 5_Q6ZMJ2-1; Most normal tissues moderate positivity. Strong in GI tract, gall bladder, adrenal gland and lymphoid cells outside the reaction center. Hepatocytes, pancreas, CNS, respiratory and squamous epithelia weak or neg. TB & Decidua weak [15]	-	6_0
SLC16A6 -8.4 ↓	Monocarboxylate transporter 7_O15403; T lymphocytes, monocytes, plasma, tonsil [19]. TB neg; decidua not available [15]	Plasma [18]	1_1
SEMA3C -8.4 ↓	Semaphorin-3C_Q99985; Peripheral blood, adipose tissue, dendritic cells, endometrium, epithelial cell lines, fibroblasts, monocytes, neutrophils, ovarian surface epithelium, plasma, skin cell lines, stromal cells, uterus [19]. TB & decidua neg [15]	Blood [19]	6_0
F11R -8.4 ↓	Junctional adhesion molecule A_Q9Y624; Moderate to strong cytoplasmic staining in majority of normal tissues. TB & Decidua strong [15]	Plasma (peptideatlas) Platelets, RBCs [22] T lymphocytes [23]; 74.1–293.2 pg/mL [24]	10_1
CFH /// CFHR1 -8.4 ↓	Complement factor H (Isoforms 1 to 2) Complement factor H-related protein 1_Q03591; Strong expression in connective tissue and plasma. TB & Decidua neg [15]	Plasma [18] 0.5 mg/mL in serum [25]	52_3
CDNA clone IMAGE:5267797 -8.6 ↓	Interleukin-1 receptor-associated kinase 4_Q9NWZ3; Moderate in many squamous tissues, strong in glandular tissue types. TB strong/Decidua moderate [15]	Plasma [18]	0_1
SLCO4A1 -8.6 ↓	Solute carrier organic anion transporter family member 4A1 (Isoforms 1 to 4)_Q96BD0-1; Granular cytoplasmic expression in selected tissues. TB moderate-weak/Decidua neg [15]	-	-
KRT14 -8.7 ↓	Keratin, type I cytoskeletal 14_P02533; Myoepithelial cells, squamous epithelium. TB & Decidua moderate [15]	Plasma [18]	11_1
MAGEB6 -8.9 ↓	Melanoma-associated antigen B6_Q8N7X4; TB strong/Decidua not available [15]	-	6_0
TNRC9 -9.0 ↓	TOX high mobility group box family member 3 (CAG trinucleotide repeat-containing gene F9 protein, Trinucleotide repeat-containing gene 9 protein)_O15405; TB & Decidua neg [15]	-	5_0
TMC4 -9.0 ↓	Transmembrane channel-like protein 4 (Isoforms 1 to 3)_Q7Z404−1 to −3; TB & Decidua neg [15]	-	3_0
ASCL2 -9.2 ↓	Achaete-scute homolog 2_Q99929 ; Cytotrophoblastic cells, T lymphocytes, placenta, tonsil [19]. Pending THPA [15]	-	-
DEPDC7 -9.4 ↓	DEP domain-containing protein 7 (Protein TR2/D15 Isoforms 1 to 2)_Q96QD5−1 to −2; Most normal tissues strong positivity. TB & Decidua strong [15]	-	5_0
RUFY3 -9.6 ↓	Protein RUFY3 (Rap2-interacting protein x, Isoforms 1 to 2)_Q7L099−1 to −2; T lymphocytes, tonsil [19]. TB & Decidua moderate [15]	-	4_0
CDNA clone IMAGE:5287025 -9.7 ↓	Cytochrome c oxidase subunit I_D9YT98; Retina, liver, brain, neurons, renal cortex, amniotic fluid, endothelial cells, high endothelial postcapillary venule, macrophages, placenta, prostate gland, tonsil [19]	Other subunits in plasma [18]	2_0
HPS3 -9.8 ↓	Hermansky-Pudlak syndrome 3 protein (Isoforms 1 and 2)_Q969F9-1; Brain, fibroblasts, heart, kidney, liver, lung, pancreatic, placental, skeletal [19]. TB moderate/Decidua strong [15]	-	13_0
LRAP -9.9 ↓	Endoplasmic reticulum aminopeptidase 2 (Leukocyte-derived arginine aminopeptidase [L-RAP]) Isoforms 1 to 4_Q6P179−1 to −4; Normal tissues weak to moderate staining. Alveolar macrophages strong. Salivary gland, pancreas, cells in CNS and parathyroid neg. TB & Decidua moderate [15]	1.00 × 10^3^ pg/mL Plasma [18]	12_1
FSTL3 -10.0 ↓	Follistatin-related protein 3 (Follistatin-like protein 3,Follistatin-related gene protein Isoforms 1 to 2)_O95633−1 to −2; Most normal tissues moderate to strong. TB moderate/Decidua strong [15]	1.20 × 10^4^ pg/mL Plasma [18]; 1.37–1.70 ng/mL 11–15 weeks [26]; 7013–55,484 pg/mL 1st trimester [27]	3_3
IGFBP1 -10.3 ↓	Insulin-like growth factor-binding protein 1_P08833; Majority of normal tissues neg. Moderate to strong in subset of basal cells in testis. TB weak/Decidua moderate to strong [15]	6.00 × 10^4^ pg/mL Plasma [18]; 24–83 ng/mL [28]; 1st and 2nd trimester; 9.34 ± 1.34 mg/L nonpregnant [29]	40_7
C6orf142; MLIP -10.7 ↓	Muscular LMNA-interacting protein_Q5VWP3; TB neg/Decidua not available [15]	-	-
SART3 -13.8 ↓	Squamous cell carcinoma antigen recognized by T-cells 3 ([SART-3, hSART-3] Tat-interacting protein of 110 kDa [Tip110] Isoform 3)_Q15020-1; B-lymphocyte derived cell lines, fibroblast cell lines, B lymphoblastoid cell lines, brain, lymphoblastoid cell lines, PBMCs, colon, embryonic cell lines, epithelial cell lines, heart, kidney cell lines, liver cells, lung, pancreatic tissue, peripheral blood leukocytes, placenta, plasma, small intestine, spleen, testicular tissue, thymus gland [19]. TB & Decidua strong [15]	Plasma [18]	15_0
Transcribed locus AFFY ID 242842_AT 14.5 ↓	Gamma-Parvin_Q9HBI0; Mainly stained moderately in glandular and transitional epithelial tissues. Lymph node moderate, hematopetic cells weak. TB weak/Decidua neg [15]	-	12_1
EPAS1 -15.3 ↓	Endothelial PAS domain-containing protein 1_Q99814; Most normal tissues moderate positivity. Strong in thyroid gland. Distal tubules stronger staining than proximal tubules. TB moderate/Decidua neg [15]	Plasma [18]	13_0
PAEP -15.6 ↓	Glycodelin ([GD], Placental protein 14 [PP14], Pregnancy-associated endometrial alpha-2 globulin [PAEG, PEG], Progestagen-associated endometrial protein, Progesterone-associated endometrial protein, Isoform 1 to 3)_P09466−1 to −3; Endometrial glands, placenta and fallopian tube distinct expression. TB moderate/Decidua neg [15]	116–870 ng/mL 1st, 30–228 ng/mL 2nd, 13–74 ng/mL 3rd trimesters [30]; 17–497 uIU/L nonpregnant [31]	17_1
MMP12 -17.2 ↓	Macrophage metalloelastase ([MME], Macrophage elastase [ME, hME], Matrix metalloproteinase-12 [MMP-12])_P39900; Most normal tissues weak to moderate. Seminal vesicle strong. Squamous epithelia, breast glands and neuronal tissues generally neg. TB moderate/Decidua neg [15]	Macrophages, basophils [19]	14_1
LAIR2 -18.9 ↓	Leukocyte-associated immunoglobulin-like receptor 2 ([LAIR-2], CD antigen CD306, Isoforms 1 and 2)_Q6ISS4-1; Synovial fluid, urine, B-lymphocyte derived cell lines, PBMCs [19]. Pending THPA [15]	1–2 mg/mL plasma 1st trimester (Our unpublished data)	2_1

* J5 score is the within dataset relative up-/down-dysregulation (↑/↓) in preeclampsia (PE) *vs.* normal CVS [7]; ** --- No UniProt Accession data 29 Apr 2011; ^†^ Number of commercially available antibodies (ABs) with human reactivity specifically applied to ELISA_ Number of ELISA kits (Biocompare www.biocompare.com/, USCN, antibodies-online) accessed 29 Apr 2011; UniProt http://www.uniprot.org/ *accessed* 9 May 2011 and 13 June 2015; THPA http://www.proteinatlas.org/ accessed 29 Apr 2011 and 13 June 2015 [19].

#### 2.1.2. MRM Assay Development

Idiosyncrasies of antibodies and the lack of analogy to oligonucleotide probes generally limit the resolution of peptide sequences [14]. These issues led us to focus on high throughput MRM based targeted proteomic assays which do not require the availability of antibodies to test for protein expression and to quantitate concentrations in maternal serum.

**Figure 1:**
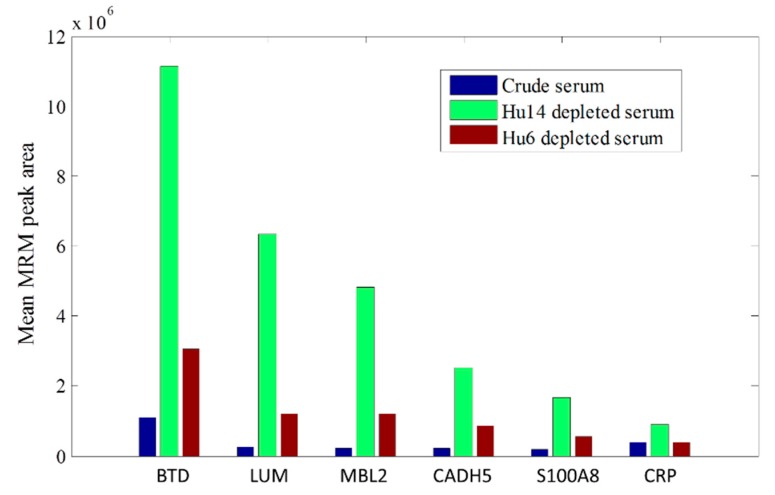
Immunodepletion performance assessment using MRM assays of selective serum proteins. Each value shown was based on the average of duplicative measurement. Largest MRM peak areas were observed with Hu14 depletion for all selective proteins. Please see Table 2 for abbreviations.

Human serum/plasma proteome is known for its large dynamic range, including the top 20 proteins which contribute to 99% of serum/plasma protein mass [14]. To increase the likelihood of detecting middle and low abundant serum proteins, we tested the effect of two different immunodepletion columns for removal of high abundant serum proteins, Agilent Human 6 Multiple Affinity Removal column (Hu6) and Agilent Human 14 Multiple Affinity Removal column (Hu14). A pooled plasma sample pooled from healthy non-pregnant female and male controls was used to compare the effect of the two different columns. Relative abundance of selective middle and low abundant serum proteins before depletion, or after depletion with Hu6 or Hu14, were measured by MRM assays. As indicated in Figure 1 and Table 2 for the results of a QC serum sample, Hu14 column out-performed Hu6 column, with higher relative abundance of these selective proteins indicated by higher MRM signals.

**Figure 2:**
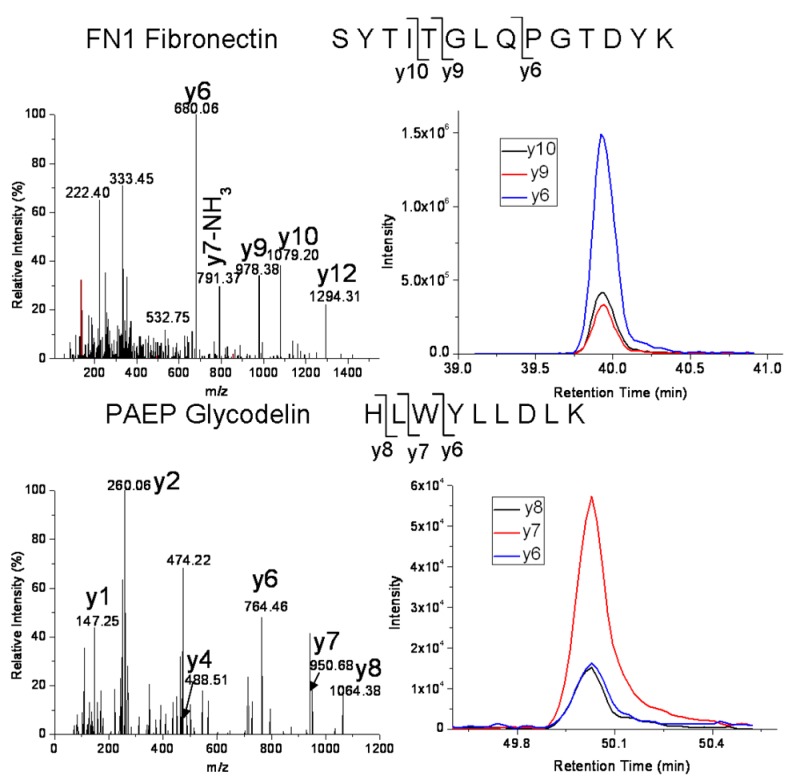
Examples of validation of peptides for two of our targeted candidate proteins by MRM-triggered MS/MS (**left** panels) and optimization of the transitions for serum MRM assays (**right** panels). The illustrated validated peptides derive from fibronectin (**top**) and glycodelin (**bottom**), respectively.

To develop MRM assays for candidate proteins of interest selected from our tissue mRNA studies, a serum sample pooled from 10 individual healthy pregnant controls was obtained. Given the superior performance of MRM assays for Hu14 depleted serum sample compared to Hu6, we applied Hu14 immunodepletion to remove the top 14 abundant serum proteins from the pooled serum sample. Tryptic peptides of Hu14 depleted serum proteins were used for the initial MRM method development. The Skyline software tool [32] was used for selection of peptide ions/peptide fragment ions (transitions), validation of MRM assays for selected peptides, and data analyses. Four proteins, fibronectin, glycodelin, protein S100-A8 and complement factor H-related protein 1 were found to have peptides with detectable MRM signals (Table 3). The validity of the developed MRM assays peptides was verified using MRM-triggered MS/MS fragmentation, as demonstrated by examples in Figure 2. The peptides were validated by observing the abundant y ions in the MS/MS spectra of the targeted peptides.

**Table 2:** List of targeted proteins and corresponding peptides and transitions for comparison of different immunodepletion columns for removal of high abundant serum proteins.

Gene	Protein	MRM Assay		Mean MRM Peak Area *^a^*		
		Peptide Sequence	Transitions	Crude Serum	Hu14 Depleted Serum	Hu6 Depleted Serum
BTD	Biotinidase	VDLITFDTPFAGR	726.4 → 1011.5, 910.4, 648.3	1.10 × 10^6^	1.11 × 10^7^	3.05 × 10^6^
LUM	Lumican	LPSGLPVSLLTLYLDNK	979.1 → 1489.8, 1293.7, 766.4	2.57 × 10^5^	6.35 × 10^6^	1.20 × 10^6^
MBL2	Mannose-binding protein C	TEGQFVDLTGNR	668.8 → 1106.6, 921.5, 774.4	2.13 × 10^5^	4.80 × 10^6^	1.19 × 10^6^
CADH5	Cadherin-5	EYFAIDNSGR	586.3 → 879.4, 732.4, 548.2	2.14 × 10^5^	2.51 × 10^6^	8.62 × 10^5^
S100A8	Protein S100-A8	LLETECPQYIR	711.4 → 1195.5, 1066.5, 836.4	1.86 × 10^5^	1.66 × 10^6^	5.42 × 10^5^
CRP	C-reactive protein	GYSIFSYATK	568.8 → 916.5, 829.4, 716.4	3.92 × 10^5^	8.91 × 10^5^	3.98 × 10^5^

*^a^* The mean MRM peak area was determined based on duplicate MRM-MS analysis.

**Table 3:** List of targeted proteins and corresponding peptides and transitions for the developed MRM assays.

Gene Name	Protein Name	Peptide Sequence	Transitions
FN1	Fibronectin isoform 1	SYTITGLQPGTDYK	772.4 → 680.3, 978.5, 1079.5
FN1	Fibronectin isoform 1	EESPLLIGQQSTVSDVPR	978.0 → 672.4, 860.4, 1173.6
PAEP	Glycodelin	HLWYLLDLK	600.8 → 764.4, 950.5, 1063.6
CFHR1	Complement factor H-related protein 1	ITCTEEGWSPTPK	753.4 → 529.3, 772.4, 901.4, 1131.5
S100A8	Protein S100-A8	LLETECPQYIR	711.4 → 836.4, 965.4, 1066.5, 1195.5

**Figure 3:**
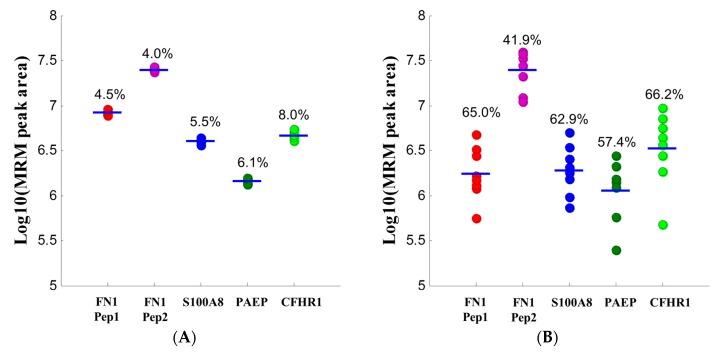
MRM peak area distribution for analyses of a pooled and eight individual serum samples. For FN-1, two peptides were quantitated (see Table 1 for protein names). Peptide 1 (Pep1) represents EESPLLIGQQSTVSDVPR and peptide 2 (Pep2) represents SYTITGLQPGTDYK, respectively. (**A**) Peak area distributions obtained in 10 replicate analyses of the pooled control sample with indicated observed CV for each; (**B**) Mean peak area distributions observed in three replicate analyses of each monitored peptide among eight individual healthy control samples. The numbers indicate the observed inter-individual variations.

We tested the reproducibility of the developed MRM assays by carrying out 10 replicate analyses of the same pooled serum sample and also the population variation by applying the assays to eight individual healthy pregnant control samples. The results are shown in Figure 3. As indicated in Figure 3A, very reproducible results were obtained in the 10 replicate analyses, with the coefficients of variation (CVs) between 4.0% and 8.0%. The results of eight individual healthy control samples are shown in Figure 3B. As expected, we observed much greater variation in these eight individual samples due to the inter-individual variations, with CVs ranging from 41.9% to 66.2%.

#### 2.1.3. Support for the Developed MRM Assays

Before investing first trimester case/control samples for further pilot studies, we examined whether the proteins detected by the developed MRM assays could be reproduced by using a second quantitative method. The maternal serum abundance of expressed protein in low volume samples was measured by ELISA for the fetoplacental glycodelin (*PAEP*).

Characteristics of the study groups for all of the pilot studies being reported are provided in Table 4. The majority of women were nulliparous at sample collection. The groups differed as expected by blood pressure criteria used to jury the preeclampsia cases and uncomplicated controls. The random selection of cases and controls by numbers of available aliquots for each subject led to a slight difference in gestational age at sampling. Based on the exploratory level of the pilot work in progress, determining that we could identify candidate proteins in both cases and controls was the goal, rather than expecting to determine differences in concentrations; thus, these small differences were not considered to be a problem.

The distribution of glycodelin abundance in cases at average 9.7 weeks of gestation and control samples at average 11.1 weeks were analyzed. This protein was measurable in both cases and controls. There was no difference between groups in glycodelin concentrations (*p* *=* 0.49). The correlation between glycodelin concentration and MRM peak area was significant (Pearson correlation 0.82; *p*-value 0.01).

**Table 4:** Study groups.

Group (*n* = 41)	Case (5)	Control (36)	*p*-Value
Parity (*n* > 0)	0	1	0.12
Gestational age at sampling (mean, SD)	9.7 (1.2)	11.1 (0.8)	0.002 *
Race (*n* not Caucasian)	0	7	1.00
BMI (mean, SD)	27.4 (7.0)	25.4 (5.8)	0.50
Smoker in pregnancy (*n*)	0	6	1.00
Average systolic blood pressure in labor (mean, SD)	145.4 (20.5)	119.9 (8.5)	0.0 *
Average diastolic blood pressure in labor (mean, SD)	89.4 (8.6)	71.4 (10.5)	0.001 *
Gestational age at delivery (mean, SD)	39.0 (1.2)	39.6 (1.6)	0.49
Birthweight (mean, SD)	2928.2 (491.6)	3357.7 (545.1)	0.10
Less than 10th percentile (*n* with SGA)	3	6	0.06

* *p* < 0.05.

#### 2.1.4. Application of MRM Assays to Case and Control Samples

Supported by the correlation between the glycodelin ELISA and MRM peak area, we went on to investigate whether the serum abundance of the four selected proteins could be determined in subjects with later preeclampsia and healthy controls. Table 5 displays the mean MRM peak area (±SD) by sample group, normalized based on the total amount of serum proteins. None of the targeted proteins demonstrated significant difference between cases and controls in this small sample set.

**Table 5:** Case-control comparisons among detected peptides.

Gene Name	Accession & Peptides	Mean (± SD)		*p*-Value (Rank Sum)
		Case	Control	
FN1	EESPLLIGQQSTVSDVPR	1.26 × 10^7^ (±5.65 × 10^6^)	1.29 × 10^7^ (±5.78 × 10^6^)	0.78
FN1	SYTITGLQPGTDYK	4.02 × 10^7^ (±1.55 × 10^7^)	4.41 × 10^7^ (±2.10 × 10^7^)	0.34
PAEP	HLWYLLDLK	2.95 × 10^6^ (±1.45 × 10^6^)	2.57 × 10^6^ (±1.35 × 10^6^)	0.74
CFHR1	ITCTEEGWSPTPK	1.01 × 10^7^ (±4.09 × 10^6^)	1.27 × 10^7^ (±6.59 × 10^6^)	0.54
S100A8	LLETECPQYIR	2.73 × 10^6^ (±1.50 × 10^6^)	2.96 × 10^6^ (±1.44 × 10^6^)	0.62

### 2.2. ELISAs with Additional Candidates

Because too few candidate peptides were detected by the MRM assays, we conducted additional ELISA pilot studies in a continuing effort to measure proteins secreted by placental/decidual tissues and encoded by the preeclampsia candidate genes [7]. ELISA assays were available for several of the protein candidates, but careful examination and consultation with others indicated many of these were poorly characterized. We limited our ELISA analyses to those assays determined to be reliable.

The serum abundance of expressed protein in low volume samples was measured by ELISA for maternal IGFBP-1 (Table 1 abbreviations) in case and control samples. Concentrations were not significantly different between preeclampsia and uncomplicated pregnancy samples in the first trimester (*p* *=* 0.18). Borderline difference was found in LAIR-2 concentrations between cases and controls (*p* *=* 0.06).

### 2.3. Immunoblotting of Candidates with Secreted Protein

With limited numbers of ELISA kits commercially available, we decided to screen for proteins in maternal circulation by immunoblotting methods for candidate proteins which other researchers had demonstrated in human circulation (Table 1). Initially, monoclonal antibodies (mAbs) were selected with the aim of increased specificity. First trimester control samples were utilized.

The results of Western immunoblots completed are displayed in Table 6. Two mAbs for LAIR-2 produced opposite results. One set of gels for fibronectin-1 produced clear bands; however, we were not able to replicate these results on a subsequent day with cases and additional control samples.

**Table 6:** Western immunoblotting results with monoclonal antibodies.

Candidate	Result	Candidate	Result
CCK	Requires RIA	MMP-12	No signal
CFH-1	No signal	S100-A8	No signal
FN-1	Signal *vs.* none	SEMA-3C	No signal
LAIR-2	Signal *vs.* none	SLC16-A6	One ab; no rhP *

* rhP recombinant human protein.

Polyclonal antibody (pAb) for MMP-12 resulted in bands at the proenzyme and active enzyme molecular weights. In an additional experiment to pilot homemade *versus* commercial buffer, no signal was obtained.

## 3. Discussion

This series of pilot studies in proteins encoded by a subset of preeclampsia candidate genes progressed from our focus on the potential for a multiplex screen with low-volume blood samples to ELISAs of individual candidates to lower sensitivity/specificity Western immunoblots. Our underlying assumption has been that the use of first trimester samples would allow for dissection of the complex disorder by mitigating issues of cause *versus* effects of the disease and labor and delivery on gene transcription and translation in second and third trimester placental tissues [33,34]. Motivated by the review of literature and database searches, we pursued the possibility of detecting candidate proteins or peptides in maternal circulation.

Development of the targeted MRM assays did identify potential candidate peptides with good coefficients of variation in pooled samples. As anticipated, much greater variability in peptide/protein abundance was observed among the eight individual samples, reflecting the inherent biological heterogeneity of individual subjects. The strong correlation between glycodelin concentrations on ELISA case-control analysis and the glycodelin MRM peak area supported the MRM assays that were developed. Ultimately, however, there were a limited number of candidate peptides detected by MRM assay. We suspect that this is likely due to the low abundance of these candidate proteins in serum. Additional steps to enrich low abundant serum proteins, in addition to removal of the top 14 abundant serum proteins, will be needed to further pursue the possibility of using multiplex MRM assays for the detection of the remaining candidate proteins. We were able to measure glycoledin and IGFBP-1 using ELISA kits with case and control samples. Exploratory analyses revealed nonsignificant differences, likely due to limited sample sizes and large inter-individual variation. To our knowledge, evaluation of glycodelin in maternal circulation is novel in the first trimester of pregnancies with and without preeclampsia. Glycodelin regulates the timing and sequencing of events in fertilization and the uterine environment for pregnancy [35]. IGFBP-1 circulates in the plasma and binds both insulin-like growth factors (IGFs) I and II, which prolongs the half-life of the IGFs and alters their interaction with cell surface receptors [35].

Issues with LAIR-2 antibodies and ELISA kit led to our basic immunoblotting to examine maternal plasma and urine. It is possible that cross-reactivity of some antibodies occurred because soluble *LAIR2* is 84% homologous with membrane-bound *LAIR1* [36]. Lebbink and colleagues [37] did not describe how dilutions were made for ELISAs or Westerns, but stated they were unable to detect LAIR-2 in plasma or sera. Although LAIR-2 protein had been detected in the urine of pregnant women [37], the genome-wide microarray analysis of CVS specimens was the first to identify the gene being expressed at the maternal-fetal interface in the placenta, where it was down-regulated in preeclampsia cases [7,11]. In a project with colleagues in the U.K., a lab-made ELISA using the mAb 1A7/FMU-LAIR-2, the same that we used in Western blots, detected no difference in LAIR-2 levels in the urine samples of women at 10–16 weeks of gestation [27]. Genomic *in situ* hybridization and protein immunohistochemical localizations were conducted in archived sections of termination tissues from pregnancies with unknown outcomes. These studies demonstrated specificity of *LAIR2* expression by the invasive first trimester extravillous trophoblasts [38] and by trophoblasts involved with remodeling the decidual spiral arterioles [39]. *LAIR2* functions in innate immunity. Under-expressed LAIR-2 may contribute to the failed remodeling of maternal spiral arteries in preeclampsia.

Reproducibility of protein data based on the limitations of antibodies was a major concern that motivated our initial efforts with LC-MS/MS. The research community’s access to commercially-produced reliable and valid antibodies has been an issue for due to variability among lots of the same antibody, nonspecific binding of extra proteins, and lack of standards for commercial production of antibodies [40,41]. In the current pilot studies, optimization of a CTAG-2 ELISA kit (MBS917872, MyBioSource, San Diego, CA, USA) was unsatisfactory and there was a dearth of available alternatives. As well, several Western immunoblots displayed no signal in candidates which had been found in circulation by other investigators. Results were inconsistent for our pilot FN-1 and MMP-12 immunoblots.

Quantitation of FN-1 and MMP-12 proteins in maternal circulation may require development of sandwich ELISAs for better sensitivity than the Western blot methods. Fibronectin functions in cell adhesion and migration [35] and encodes a large molecule with 20 isoforms [42] which may account for different peptides detected by LC-MS/MS. Other groups demonstrated that glycosylated fibronectin was higher in each trimester of preeclampsia compared with uncomplicated pregnancies [43], and was also elevated in the first trimester of pregnancies developing gestational diabetes [44]. We were most interested in the embryonic, cellular isoform containing the EDB domain [42], particularly because the microarray probes included transcripts in this domain [7]. MMP-12 functions in cell adhesion, extracellular matrix remodeling, and elastin degradation. Harris and colleagues also identified *MMP12* as one of the most highly expressed protease genes in isolated first trimester extravillous trophoblasts and found that it degraded elastin during vascular remodeling in the placenta [45].

In contrast, we were able to reliably quantify FSTL-3 in samples from cases and controls [46]. Plasma concentrations differed across gestation by trimester in lean controls (first to second trimester, *p* *=* 0.01; second to third trimester, *p* *=* 0.0001) and obese women with preeclampsia (first to third trimester, *p* *=* 0.0002; second to third trimester, *p* *=* 0.007), but did not differ significantly by trimester in obese controls (*p* > 0.05) [46]. Among the three body mass index-based study groups, FSTL-3 concentration was affected by preeclampsia but not by adiposity measures which were determined by bioelectric impedance analysis [46]. When FSTL-3 was elevated in the second trimester, the odds of preeclampsia was increased 3.15 fold (odds ratio 3.15; 95% confidence interval 1.19–8.36; *p* *=* 0.02) [46]. *FSTL3* encodes a secreted glycoprotein that antagonizes members of the transforming growth factor β superfamily of growth and differentiation factors [47]. *FSTL3* functions in signal transduction of activin A [48] which was elevated in preeclampsia [49]. Circulating FSTL-3 was also found to be higher in the first trimester of gestational diabetes [50]. Specificity to preeclampsia prediction or detection will likely require FSTL-3 levels to be combined with other clinical, demographic, and molecular biomarkers.

Criteria such as early *versus* late onset of disease in cases, and matching with controls by gestational age were not implemented for the currently reported proteomic studies because these pilots aimed to detect presence *versus* absence of candidate proteins along with feasibility of quantitation. It is noteworthy that all donors of the utilized samples delivered at term, suggesting late onset preeclampsia; however, assumptions regarding the roles of maternal predisposition to preeclampsia *versus* feto-placental pathogenesis and gestational age of onset remain to be determined and tested.

## 4. Experimental Section

### 4.1. Samples

De-identified banked frozen blood samples from women who went on to develop the juried outcomes were used for the pilot studies reported in this paper. The IRB-approved Pregnancy Exposures and Preeclampsia Prevention (PEPP) studies of pregnant women, who enrolled at <21 weeks and were followed through pregnancy and the postpartum visit, provided first trimester samples from among those donated by 2812 women recruited from 1997 to 2006 in PEPP1 and 601 enrolled between 2002 and 2006 in PEPP2. Gestational age was determined by last menstrual period and ultrasound as abstracted from review of medical records. Data were not collected for gestational age at onset of disease during these studies.

Inclusion criteria for the types of samples were: preeclampsia defined as new onset of hypertension and proteinuria after 20 weeks of gestation with blood pressure ≥140 and/or 90 on at least two occasions at least six hours apart, ≥300 mg of protein in a 24 h urine or 1+ urine on a catheterized urine specimen, 2+ on a random specimen or a protein:creatinine ratio ≥0.3 [51]; and control defined by normotensive, nonproteinuric, uncomplicated pregnancies. Exclusion criteria were samples of women with pregnancy complications, non-obstetric medical complications, and gestational age >13 weeks.

Clinical and demographic variables (Table 4) were compared between cases and controls by using chi square analyses, Fisher Exact Tests, and *t*-tests as distributionally appropriate with significance set at *p* ≤ 0.05.

### 4.2. MRM Assays

#### 4.2.1. Database Search

Reliable proteomic literature and databases were searched in order to query indications for the feasibility of detecting secreted proteins from the preeclampsia candidate genes in maternal circulation. PubMed, OMIM, Uniprot were searched for each candidate. The original searches that motivated our studies in 2011 are presented with current updates from The Human Protein Atlas (THPA) [15] for candidates that had been pending at that time.

#### 4.2.2. Immunoaffinity Removal of High Abundant Serum Proteins

High abundant serum proteins were removed using Agilent Multiple Affinity Removal spin cartridges/columns (Agilent Technologies, Palo Alto, CA, USA) according to manufacturer’s protocol. Two types of depletion scenario were evaluated: depletion of the top six most abundant serum proteins ((albumin, IgG, antitrypsin, IgA, transferrin and haptoglobin) and depletion of the top 14 most abundant serum proteins (albumin, IgG, antitrypsin, IgA, transferrin, haptoglobin, fibrinogen, alpha-2-macroglobulin, alpha-1-acid glycoprotein, IgM, apolipoprotein AI, apolipoprotein AII, complement C3, and transthyretin). The flow-through fractions containing the medium/low abundant serum protein were buffer exchanged into 50mM NH4HCO3 using Amicon Ultra 5000 MWCO devices (Millipore, Bedford, MA, USA). The micro BCA protein assay kit (Pierce, Rockford, IL, USA) was used to determine total protein concentrations prior to in solution trypsin digestion.

#### 4.2.3. MRM Assay Development

In an initial pilot effort, we developed MRM serum assays for six peptides from five expressed and circulating proteins expressed by the corresponding five genes included among our targeted 36 gene candidates (Table 3). We utilized the Skyline software tool [32] for this process including MRM method development, validation of targeted peptides, and data analyses of our MRM raw data. For the initial step of MRM method development, we applied the following criteria for candidate peptide selection: (1) peptides were unique to the proteins expressed by the targeted gene; (2) peptides were doubly charged containing 6–20 amino acids; (3) no missed or ragged cleavages in the selected peptides; and (4) the peptides had been previously observed in shotgun proteomics analyses of human plasma based on the information provided in the human plasma build of PeptideAtlas [52].

Serum proteins after immunodepletion were trypsin digested and the resultant tryptic peptides were analyzed by LC-MRM similarly as described [53]. In brief, the tryptic peptides were then analyzed using a Quantum ULTRA (ThermoFisher Scientific, Waltham, MA, USA) coupled with a nanoflow Dionex Ultimate 3000 liquid chromatography system using 2% acetonitrile in 0.1% formic acid as solvent A and 100% acetonitrile in 0.1% formic acid as solvent B. Tryptic digests from 10 μg post-MARS14 depletion serum proteins were resuspended in 20 μL 0.1% TFA and analyzed in triplicate (6 μL for each injection). Nano LC separation was performed using a homemade Jupiter C18 fused silica capillary column (75 µm I.D. × 360 µm O.D. × 20 cm-long) (Polymicro Technologies, Phoenix, AZ, USA). The peptides were eluted at 350 nL/min with a gradient of 0%–35% solvent B for 35 min, 35%–95% solvent B for 3 min, 95% solvent B for 10 min. The collision energies were calculated using the standard equation CE = 0.034 × *m*/*z* + 3.314. FHWM (full width at half maximum) was set to be 0.7 Da for Q1 and Q3. The dwell time at each transition was 10 ms and the width of detection window for each transition was 1 *m*/*z*.

In the MRM assay development, 5–8 transitions corresponding to the y-ion series for all of the selected peptides were monitored. The peptides with co-eluted transitions were validated by their product-ion mass spectra acquired by MRM-triggered MS/MS experiments. After validation, the transitions of the validated peptides were optimized based on the intensity of the product ions in the MS/MS spectra and then multiplexed in a single MRM analysis. To achieve high sensitivity and specificity for the MRM assays, the most abundant y ions were selected and used as transitions during the MRM analyses.

#### 4.2.4. Validation Studies

We then evaluated the analytical performance of the MRM assays first in multiple analyses of a pooled serum sample derived from serum samples (0.52–16.00 μL) of 10 healthy control women. This pooled sample was analyzed in 10 replicates in order to test the reproducibility of the developed MRM assays for each peptide. Next, in order to evaluate the relative peptide abundance among individual serum samples, triplicate analyses of eight individual healthy control samples were also performed and averaged. Coefficients of variation were analyzed.

To evaluate the maternal serum abundance of expressed protein of candidate genes in low volume samples, in order to validate the developed MRM assays, we quantitated fetoplacental glycodelin levels (Table 1) using a commercially available human ELISA kit (ABIN418389, antibodies-online.com, Atlanta, GA, USA) according to manufacturers’ instructions. Serum from five preeclampsia and 25 controls were analyzed. Each sample was measured in duplicate and results averaged. Mann-Whitney U and Pearson correlation with respective MRM peak area values were conducted with *p*-value ≤0.05 for significance.

### 4.3. ELISAs with Additional Candidates

ELISAs were performed with commercially available kits according to manufacturers’ instructions. Each sample was measured in duplicate. LAIR-2 (ABIN417567; USCN antibodies-online.com, Atlanta, GA, USA) was conducted in five preeclampsia and 17 control samples. Maternal biomarker IGFBP-1 (ELH-IGFBP1-00, 1; RayBiotech, Norcross, GA, USA) was measured in serum from five preeclampsia and 25 controls. Mann-Whitney U and *t*-tests were conducted with the averaged concentrations from each sample (*p* ≤ 0.05).

### 4.4. Immunoblotting

Western immunoblots for LAIR-2 were piloted in plasma and urine, and all other candidates in serum and plasma of multiparous controls who consented to participate in the PEPP1 project. Inclusion/exclusion criteria were defined as in Section 4.1.

The antibodies and recombinant human proteins used for immunoblotting are provided in Table 7.

**Table 7:** Antibodies and recombinant proteins utilized.

Candidate	Catalog Number, Company; Site	Candidate	Catalog Number, Company; Site
CFH-1	MAB4779, Clone 556317 mAb & 4779-FH-050 rhCFH-1, R&D Systems, Minneapolis, MN	MMP-12	MAB919 mAb, AF917 pAb & WBC019 rhMMP-12, R&D Systems
FN-1	ab154211 mAb, Cambridge, MA; #161-0375 rhFN-1, Bio Rad Hercules, CA	S100-A8	MAB4570, Clone 749916 mAb & 8226-S8-050, rhS100A8/S100A9 heterodimer, R&D Systems
LAIR-2	1A7/FMU-LAIR-2 mAb gift of Boquan Jin; MAB2665 mAb & 2665-LR-050 rhLAIR-2, R&D Systems	SEMA-3C	MAB1728 Clone 238835 mAb & 5570-S3-050 rhSemaphorin 3C Fc Chimera; R&D Systems

Western blot procedures were followed according to the manufacturers’ methods. More specific details are available upon request from the corresponding author. Based on the molecular weight of the protein of interest, the percentage of acrylamide in the separating gel varied, as did the gel to membrane transfer times. Antibodies were diluted in homemade BLOTTO based on R&D Buffer Group 1 [54]. After semi-dry transfer, the membrane was briefly soaked in methanol, allowed to air dry, rewet in methanol, followed with water before blocking and treating with the primary antibody overnight at 4 °C. Gels were run with undiluted urine or diluted plasma to avoid overloading the gel.

Western blots were evaluated semi-quantitatively by presence or absence of signal per lane and relative strength with and without rh-protein spikes in biofluids.

## 5. Conclusions

The pilot studies conducted thus far to detect proteins encoded by the preeclampsia candidate genes (Table 1) [7] in maternal circulation of early pregnancy began with the goal of multiplexing specific peptides. The mass spectrometry methods for detection of targeted candidates led to robust MRM assays, but were determined to be too costly for the few detected, low abundant peptides. Continued effort will need to optimize available ELISA kits, antibodies, and recombinant proteins and to develop sandwich ELISAs. Further antibody-based studies of proteins from the discovery-based system of 36 candidate genes obtained from maternal and fetal tissues at the time of spiral artery remodeling may translate to signals of susceptibility to, or protection from, preeclampsia [7,34].
